# Use of a Hysteroscopic Tissue Removal System for Revision of Cesarean Scar Isthmocele: A Case Report

**DOI:** 10.1155/crog/8858077

**Published:** 2025-12-15

**Authors:** Shahryar K. Kavoussi, Amy S. Esqueda, Krista London, Sarah M. Compton, Ellen Jatinen, Kelsey Cathcart

**Affiliations:** ^1^ Department of Reproductive Endocrinology & Infertility, Austin Fertility & Reproductive Medicine/Westlake IVF, Austin, Texas, USA

**Keywords:** cesarean section, hysteroscopy, isthmocele, morcellation, niche

## Abstract

Cesarean scar isthmocele (CSI) is a defect in the anterior myometrial wall of the uterine isthmus, at the site of a previous cesarean scar, and can be associated with menorrhagia in the form of prolonged menstrual bleeding or spotting, dysmenorrhea, and/or secondary subfertility. We report a case of hysteroscopic morcellation of CSI with a subsequent successful pregnancy. A 37‐year‐old female with menorrhagia since cesarean delivery and secondary subfertility, with midcycle transvaginal sonogram (TVS) showing a CSI with associated endometrial cavity fluid (ECF) as well as a small endometrial polyp, underwent hysteroscopic revision of the CSI and polypectomy via a tissue removal system. Postoperatively, menorrhagia had resolved, and follow‐up midcycle TVS imaging showed no ECF. The patient spontaneously conceived 3 months after surgery and achieved a successful pregnancy. The use of a hysteroscopic tissue removal system is a feasible approach to the revision of CSI. Larger studies are necessary in order to determine efficacy for fertility patients.

## 1. Introduction

Due to the increased prevalence of cesarean delivery (CD) worldwide, cesarean scar isthmocele (CSI) is being observed and diagnosed more often, particularly in the setting of menorrhagia in the form of prolonged menstrual bleeding or spotting, dysmenorrhea, and secondary subfertility [1]. CSI is a defect in the anterior myometrial wall of the uterine isthmus, at the site of a previous cesarean scar, and has been reported to occur in 56%–84% of CD [2, 3]. Women with CSI can be asymptomatic [4]; however, CSI can be associated with symptoms such as menorrhagia in the form of prolonged menstrual bleeding or spotting as well as dysmenorrhea [5–7]. In addition, CSI can be diagnosed in women with secondary subfertility [8], with a potential sonographic finding of significant fluid within the CSI which, in some cases, may track upstream into the endometrial cavity, thereby creating endometrial cavity fluid (ECF) at midcycle. The components of fluid within CSI can include accumulated menstrual blood and mucus.

Surgical treatment of CSI has been shown to improve symptoms as well as fertility [7]. The modalities to surgically correct CSI include hysteroscopic resection, laparoscopic repair, vaginal approach, or a combination of hysteroscopic and laparoscopic approaches [9]. The hysteroscopic route has been reported to be most cost‐effective [10], and methods described have included the use of resectoscopes with cutting loops as well as roller ball diathermy after resection [11–14]. To our knowledge, this is the first report of the use of a hysteroscopic tissue removal system for revision of CSI, with a subsequent successful spontaneous pregnancy outcome.

## 2. Case Presentation

**Figure 1:**
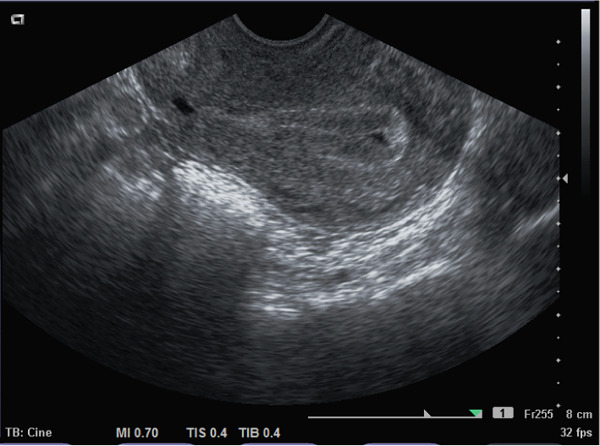
Transvaginal sonogram image at midcycle showing cesarean section isthmocele (CSI) and endometrial cavity fluid.

**Figure 2:**
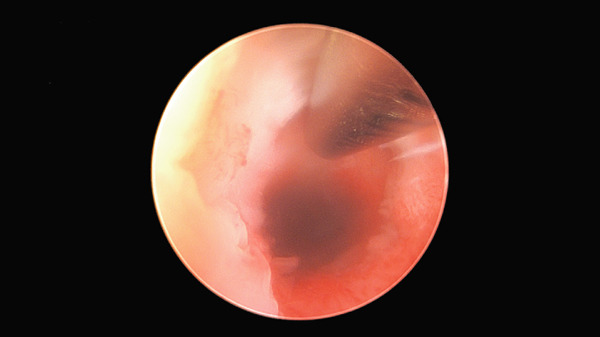
Hysteroscopic revision of CSI via TruClear™ hysteroscopic tissue removal system.

**Figure 3:**
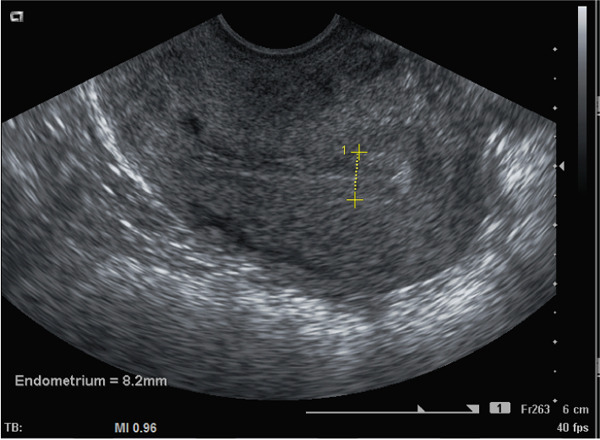
Postoperative transvaginal sonogram image at midcycle after hysteroscopic revision of CSI showing a trilaminar endometrial lining and no endometrial cavity fluid.

The patient provided informed consent for publication of this case report. A 37‐year‐old Gravida 1 Para 1 presented with secondary subfertility for 6 months. She reported having had CD 2 years earlier and, ever since the delivery, has had prolonged menstrual flow, with 7 days of full flow followed by spotting until the day of ovulation around Day 14 of her cycle. Transvaginal sonogram (TVS) on Cycle Day 12 showed an obvious CSI with fluid which tracked up into the endometrial cavity with an oval‐shaped ECF formation (Figure 1) which measured 6.4 × 1.8 mm in two dimensions and a right‐sided fundal echogenic focus measuring 6.5 mm, consistent with an endometrial polyp. Hysteroscopic surgery showed the CSI at the level of the lower uterine segment as well as a right‐sided small endometrial polyp at the right cornual region. The TruClear™ soft tissue shaver mini hysteroscopic tissue removal system (Medtronic) was used to revise the distal and proximal aspects of the CSI (Figure 2) after polypectomy had been performed. Postoperative Midcycle Day 12 TVS showed an 8.4 mm trilaminar endometrial lining without ECF (Figure 3). The next month, the patient had a 12.6 mm trilaminar endometrial lining without ECF. The patient achieved a spontaneous pregnancy 3 months after surgery which resulted in a singleton live birth.

## 3. Discussion

Revision of the CSI via morcellation with a hysteroscopic tissue removal system resulted in improvement of menorrhagia as well as the achievement of a spontaneous conception which was productive of live birth. Although there is no strict cutoff regarding features of CSI or minimal overlying myometrial thickness criteria for feasibility of a hysteroscopic approach resulting in adequate resultant site integrity after CSI resection, in order to minimize the risks of uterine rupture and bladder injury, previous studies have suggested that preoperative residual myometrial thickness should be at least 2 mm [10], greater than 3.5 mm in patients who desire to preserve fertility, and greater than 2.5 mm in those without a desire for future fertility [15]. A case report describing the hysteroscopic morcellation of a cesarean section scar ectopic pregnancy has been reported [16]; however, to our knowledge, this is the first report of hysteroscopic morcellation of a CSI, resulting in a successful pregnancy outcome. Although large studies are needed to determine the effects of hysteroscopic CSI revision via the use of a tissue removal system on fertility, this case report illustrates the feasibility of a successful spontaneous pregnancy after such an approach.

## Disclosure

S.K.K., A.S.E., K.L., S.M.C., E.J., and K.C. contributed to and approved the final version of the manuscript.

## Conflicts of Interest

S.K.K. has received honoraria from Medtronic. A.S.E., K.L., S.M.C., E.J., and K.C. have no conflict of interest.

## Author Contributions

S.K.K. conceptualized the study and drafted the manuscript.

## Funding

No funding was received for this manuscript.

## Data Availability

The data that support the findings of this study are available from the corresponding author upon reasonable request.
